# Bis(2-phenyl-1*H*-imidazole-κ*N* ^3^)silver(I) nitrate

**DOI:** 10.1107/S1600536809051459

**Published:** 2009-12-04

**Authors:** Shuang Han, Wan-Cheng Li, Dao-Cheng Xia

**Affiliations:** a College of Chemistry, Yuncheng University, Yuncheng 044000, People’s Republic of China; b State Key Laboratory of Integrated Optoelectronics, Jilin University, Changchun 130021, People’s Republic of China

## Abstract

The asymmetric unit of the title compound, [Ag(C_9_H_8_N_2_)_2_]NO_3_, contains one complete [Ag(C_9_H_8_N_2_)_2_]^+^ cation and two half-cations (with the other halves generated through inversion) and two NO_3_^−^ anions. Each Ag^I^ ion shows a linear AgN_2_ coordination. The ions are linked by N—H⋯O hydrogen bonds.

## Related literature

For general background to 2-phenyl­imidazole, see: Liu *et al.* (2008 ▶); Yang *et al.* (2008 ▶).
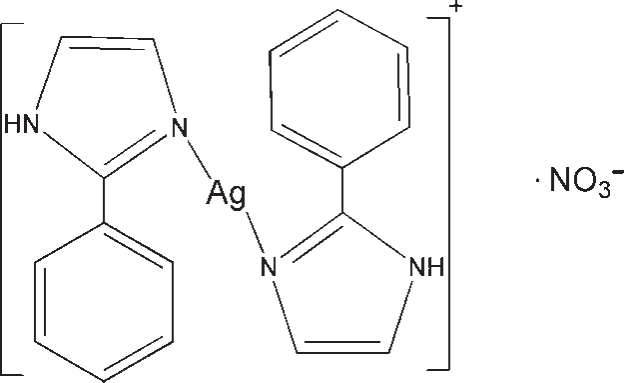


## Experimental

### 

#### Crystal data

[Ag(C_9_H_8_N_2_)_2_]NO_3_
*M* *_r_* = 458.23
Triclinic, 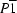

*a* = 9.209 (5) Å
*b* = 9.274 (5) Å
*c* = 23.137 (5) Å
α = 88.307 (5)°
β = 80.976 (5)°
γ = 72.369 (5)°
*V* = 1859.5 (15) Å^3^
*Z* = 4
Mo *K*α radiation
μ = 1.11 mm^−1^
*T* = 293 K
0.31 × 0.28 × 0.22 mm

#### Data collection

Oxford Diffraction Gemini R Ultra diffractometer
Absorption correction: multi-scan (*CrysAlis*; Oxford Diffraction, 2006 ▶) *T*_min_ = 0.62, *T*_max_ = 0.86
14127 measured reflections
8475 independent reflections
4372 reflections with *I* > 2σ(*I*)
*R*_int_ = 0.029

#### Refinement

*R*[*F* ^2^ > 2σ(*F* ^2^)] = 0.029
*wR*(*F* ^2^) = 0.079
*S* = 0.80
8475 reflections
490 parameters
H-atom parameters constrained
Δρ_max_ = 0.28 e Å^−3^
Δρ_min_ = −0.43 e Å^−3^

### 

Data collection: *CrysAlis* (Oxford Diffraction, 2006 ▶); cell refinement: *CrysAlis*; data reduction: *CrysAlis*; program(s) used to solve structure: *SHELXS97* (Sheldrick, 2008 ▶); program(s) used to refine structure: *SHELXL97* (Sheldrick, 2008 ▶); molecular graphics: *SHELXTL* (Sheldrick, 2008 ▶); software used to prepare material for publication: *SHELXTL*.

## Supplementary Material

Crystal structure: contains datablocks global, I. DOI: 10.1107/S1600536809051459/ci2975sup1.cif

Structure factors: contains datablocks I. DOI: 10.1107/S1600536809051459/ci2975Isup2.hkl

Additional supplementary materials:  crystallographic information; 3D view; checkCIF report

## Figures and Tables

**Table 1:** Hydrogen-bond geometry (Å, °)

*D*—H⋯*A*	*D*—H	H⋯*A*	*D*⋯*A*	*D*—H⋯*A*
N2—H2⋯O6^i^	0.86	2.03	2.893 (3)	178
N3—H3⋯O5^ii^	0.86	1.95	2.812 (3)	178
N6—H6⋯O2^iii^	0.86	1.99	2.846 (3)	173
N7—H7⋯O1^iv^	0.86	1.95	2.812 (3)	177

Symmetry codes: (i) 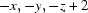; (ii) 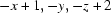; (iii) 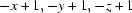; (iv) 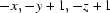.

**Fig. 1.**
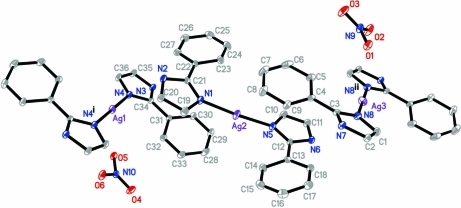
The molecular structure of the title compound, showing the atomic numbering scheme. Displacement ellipsoids are drawn at the 30% probability level. [Symmetry codes: (i) -x, -y, 2 - z; (ii) 1 - x, 1 - y, 1 -z]

## supplementary crystallographic information

### Comment

2-Phenylimidazole, as an important N-containing ligand with excellent coordinating abilities and fruitful aromatic systems, have been extensively used to build supramolecular architectures (Liu *et al.*, 2008; Yang *et al.*, 2008). We report here the synthesis and structure of the title compound, namely, [Ag(C_9_H_8_N_2_)_2_]^.^2NO_3_ (I)

The asymmetric unit contains one complete and two halves of centrosymmetric [Ag(C_9_H_8_N_2_)_2_]^+^ cations and two NO_3_^-^ anions. Atoms Ag1 and Ag3 lie on inversion centres. Each Ag^I^ ion in the title salt shows a linear coordination. The N—Ag—N angle is exactly 180° (by virtue of the inversion symmetry) for two cations lying across inversion centres and 175.94 (11)° for the cation on general position.

In the crystal structure, the ionic units are linked through N—H···O hydrogen bonds (Table 1)

### Experimental

Silver nitrate (0.5 mmol, 0.085 g) and 2-phenylimidazole (0.5 mmol, 0.041 g) in water (20 mmol) was heated at 435 K for 2 d. Then the mixture was slowly cooled to room temperature to obtain colourless single crystals of the title compound (yield: 39%).

### Refinement

H atoms were positioned geometrically (N–H = 0.86 Å and C–H = 0.93 Å) and refined as riding, with *U*_iso_(H) = 1.2*U*_eq_(carrier).

### Crystal data

**Table:**

[Ag(C_9_H_8_N_2_)_2_]NO_3_	*Z* = 4
*M**_r_* = 458.23	*F*(000) = 920
Triclinic, *P*1	*D*_x_ = 1.637 Mg m^−^^3^
Hall symbol: -P 1	Mo *K*α radiation, λ = 0.71073 Å
*a* = 9.209 (5) Å	Cell parameters from 8475 reflections
*b* = 9.274 (5) Å	θ = 3.0–29.1°
*c* = 23.137 (5) Å	µ = 1.11 mm^−^^1^
α = 88.307 (5)°	*T* = 293 K
β = 80.976 (5)°	Block, colourless
γ = 72.369 (5)°	0.31 × 0.28 × 0.22 mm
*V* = 1859.5 (15) Å^3^	

### Data collection

**Table:**

Oxford Diffraction Gemini R Ultra diffractometer	8475 independent reflections
Radiation source: fine-focus sealed tube	4372 reflections with *I* > 2σ(*I*)
graphite	*R*_int_ = 0.029
Detector resolution: 10.0 pixels mm^-1^	θ_max_ = 29.1°, θ_min_ = 1.8°
ω scan	*h* = −10→11
Absorption correction: multi-scan (CrysAlis; Oxford Diffraction, 2006)	*k* = −9→11
*T*_min_ = 0.62, *T*_max_ = 0.86	*l* = −23→28
14127 measured reflections	

### Refinement

**Table:**

Refinement on *F*^2^	Primary atom site location: structure-invariant direct methods
Least-squares matrix: full	Secondary atom site location: difference Fourier map
*R*[*F*^2^ > 2σ(*F*^2^)] = 0.029	Hydrogen site location: inferred from neighbouring sites
*wR*(*F*^2^) = 0.079	H-atom parameters constrained
*S* = 0.80	*w* = 1/[σ^2^(*F*_o_^2^) + (0.04*P*)^2^] where *P* = (*F*_o_^2^ + 2*F*_c_^2^)/3
8475 reflections	(Δ/σ)_max_ = 0.002
490 parameters	Δρ_max_ = 0.28 e Å^−^^3^
0 restraints	Δρ_min_ = −0.43 e Å^−^^3^

### Special details

**Table:**

Geometry. All e.s.d.'s (except the e.s.d. in the dihedral angle between two l.s. planes) are estimated using the full covariance matrix. The cell e.s.d.'s are taken into account individually in the estimation of e.s.d.'s in distances, angles and torsion angles; correlations between e.s.d.'s in cell parameters are only used when they are defined by crystal symmetry. An approximate (isotropic) treatment of cell e.s.d.'s is used for estimating e.s.d.'s involving l.s. planes.
Refinement. Refinement of *F*^2^ against ALL reflections. The weighted *R*-factor *wR* and goodness of fit *S* are based on *F*^2^, conventional *R*-factors *R* are based on *F*, with *F* set to zero for negative *F*^2^. The threshold expression of *F*^2^ > σ(*F*^2^) is used only for calculating *R*-factors(gt) *etc*. and is not relevant to the choice of reflections for refinement. *R*-factors based on *F*^2^ are statistically about twice as large as those based on *F*, and *R*- factors based on ALL data will be even larger.

### Fractional atomic coordinates and isotropic or equivalent isotropic displacement parameters (Å^2^)

**Table:**

	*x*	*y*	*z*	*U*_iso_*/*U*_eq_	
C1	0.2039 (4)	0.7556 (4)	0.48467 (13)	0.0638 (9)	
H1	0.2611	0.8103	0.4622	0.077*	
C2	0.0492 (4)	0.7985 (3)	0.49731 (12)	0.0594 (8)	
H2A	−0.0197	0.8856	0.4853	0.071*	
C3	0.1453 (3)	0.5807 (3)	0.53886 (11)	0.0468 (7)	
C4	0.1492 (3)	0.4477 (3)	0.57426 (12)	0.0519 (7)	
C5	0.2697 (4)	0.3145 (4)	0.56355 (15)	0.0728 (10)	
H5	0.3513	0.3085	0.5335	0.087*	
C6	0.2676 (6)	0.1892 (4)	0.5984 (2)	0.1029 (15)	
H6A	0.3488	0.1000	0.5917	0.123*	
C7	0.1483 (7)	0.1965 (5)	0.6419 (2)	0.1033 (16)	
H7A	0.1477	0.1117	0.6643	0.124*	
C8	0.0300 (5)	0.3259 (5)	0.65292 (16)	0.0858 (12)	
H8	−0.0514	0.3298	0.6828	0.103*	
C9	0.0301 (4)	0.4525 (4)	0.61976 (13)	0.0642 (9)	
H9	−0.0504	0.5417	0.6281	0.077*	
C10	0.5291 (4)	0.3440 (4)	0.68802 (15)	0.0770 (10)	
H10	0.5854	0.2473	0.6976	0.092*	
C11	0.5840 (4)	0.4361 (4)	0.65051 (14)	0.0703 (9)	
H11	0.6833	0.4156	0.6297	0.084*	
C12	0.3411 (3)	0.5502 (4)	0.68619 (11)	0.0531 (8)	
C13	0.1928 (3)	0.6666 (4)	0.69513 (12)	0.0535 (8)	
C14	0.0864 (4)	0.6708 (4)	0.74507 (13)	0.0675 (9)	
H14	0.1112	0.5999	0.7741	0.081*	
C15	−0.0549 (4)	0.7784 (5)	0.75202 (16)	0.0831 (11)	
H15	−0.1255	0.7793	0.7856	0.100*	
C16	−0.0939 (4)	0.8843 (5)	0.71036 (19)	0.0888 (11)	
H16	−0.1902	0.9570	0.7156	0.107*	
C17	0.0100 (4)	0.8830 (4)	0.66062 (16)	0.0768 (10)	
H17	−0.0162	0.9550	0.6321	0.092*	
C18	0.1526 (4)	0.7756 (4)	0.65282 (13)	0.0629 (8)	
H18	0.2225	0.7756	0.6191	0.076*	
C19	−0.0449 (4)	0.2333 (4)	0.83247 (15)	0.0759 (10)	
H19	−0.1031	0.3343	0.8314	0.091*	
C20	−0.0945 (4)	0.1228 (4)	0.86113 (13)	0.0665 (9)	
H20	−0.1908	0.1336	0.8832	0.080*	
C21	0.1440 (3)	0.0264 (4)	0.81694 (11)	0.0513 (7)	
C22	0.2921 (3)	−0.0865 (4)	0.79868 (11)	0.0513 (7)	
C23	0.3918 (4)	−0.0679 (4)	0.74913 (12)	0.0705 (9)	
H23	0.3614	0.0170	0.7263	0.085*	
C24	0.5334 (4)	−0.1714 (5)	0.73331 (16)	0.0876 (12)	
H24	0.5984	−0.1561	0.7003	0.105*	
C25	0.5795 (4)	−0.2975 (5)	0.76602 (19)	0.0915 (12)	
H25	0.6758	−0.3679	0.7552	0.110*	
C26	0.4839 (4)	−0.3200 (4)	0.81467 (17)	0.0835 (11)	
H26	0.5155	−0.4061	0.8368	0.100*	
C27	0.3413 (4)	−0.2161 (4)	0.83117 (14)	0.0657 (9)	
H27	0.2772	−0.2327	0.8643	0.079*	
C28	0.3155 (4)	0.3721 (4)	0.88477 (16)	0.0765 (10)	
H28	0.3069	0.4670	0.8689	0.092*	
C29	0.4421 (4)	0.2525 (4)	0.86556 (14)	0.0686 (9)	
H29	0.5199	0.2668	0.8372	0.082*	
C30	0.4548 (3)	0.1094 (3)	0.88841 (12)	0.0566 (8)	
H30	0.5404	0.0278	0.8751	0.068*	
C31	0.3391 (3)	0.0887 (3)	0.93131 (11)	0.0431 (7)	
C32	0.2134 (3)	0.2126 (3)	0.94971 (12)	0.0564 (8)	
H32	0.1349	0.2003	0.9782	0.068*	
C33	0.2016 (4)	0.3536 (4)	0.92696 (15)	0.0708 (9)	
H33	0.1166	0.4358	0.9402	0.085*	
C34	0.3518 (3)	−0.0615 (3)	0.95527 (11)	0.0417 (6)	
C35	0.4571 (3)	−0.2971 (3)	0.97852 (14)	0.0644 (9)	
H35	0.5294	−0.3899	0.9831	0.077*	
C36	0.3034 (3)	−0.2594 (3)	0.99545 (13)	0.0617 (8)	
H36	0.2506	−0.3234	1.0139	0.074*	
O1	0.2835 (2)	0.3113 (3)	0.41706 (10)	0.0778 (7)	
O2	0.5143 (3)	0.1699 (3)	0.41170 (11)	0.0944 (8)	
O3	0.3276 (3)	0.0750 (3)	0.43228 (14)	0.1103 (10)	
O4	0.1720 (3)	0.3963 (3)	1.08721 (12)	0.0926 (8)	
O5	0.2200 (2)	0.1559 (2)	1.09430 (9)	0.0675 (6)	
O6	−0.0137 (2)	0.2969 (3)	1.10375 (11)	0.0854 (8)	
Ag1	0.0000	0.0000	1.0000	0.05936 (11)	
Ag2	0.23738 (3)	0.30005 (3)	0.759780 (12)	0.08314 (12)	
Ag3	0.5000	0.5000	0.5000	0.07317 (13)	
N1	0.1033 (3)	0.1731 (3)	0.80549 (10)	0.0642 (7)	
N2	0.0241 (3)	−0.0054 (3)	0.85106 (10)	0.0574 (7)	
H2	0.0239	−0.0931	0.8641	0.069*	
N3	0.4853 (2)	−0.1724 (2)	0.95343 (9)	0.0512 (6)	
H3	0.5747	−0.1656	0.9387	0.061*	
N4	0.2370 (2)	−0.1122 (3)	0.98131 (10)	0.0518 (6)	
N5	0.3783 (3)	0.4142 (3)	0.70973 (11)	0.0650 (7)	
N6	0.4661 (3)	0.5638 (3)	0.64922 (10)	0.0579 (7)	
H6	0.4693	0.6415	0.6284	0.069*	
N7	0.0144 (3)	0.6876 (3)	0.53138 (10)	0.0513 (6)	
H7	−0.0771	0.6863	0.5458	0.062*	
N8	0.2649 (2)	0.6190 (3)	0.50983 (10)	0.0574 (6)	
N9	0.3743 (3)	0.1830 (3)	0.42040 (11)	0.0659 (7)	
N10	0.1257 (3)	0.2861 (3)	1.09532 (10)	0.0595 (7)	

### Atomic displacement parameters (Å^2^)

**Table:**

	*U*^11^	*U*^22^	*U*^33^	*U*^12^	*U*^13^	*U*^23^
C1	0.058 (2)	0.064 (2)	0.069 (2)	−0.0259 (18)	0.0022 (16)	0.0091 (17)
C2	0.059 (2)	0.045 (2)	0.074 (2)	−0.0155 (16)	−0.0115 (15)	0.0133 (16)
C3	0.0399 (17)	0.0372 (18)	0.0618 (17)	−0.0096 (14)	−0.0072 (13)	−0.0040 (14)
C4	0.0543 (19)	0.040 (2)	0.0655 (18)	−0.0133 (16)	−0.0252 (15)	0.0029 (15)
C5	0.071 (2)	0.052 (2)	0.093 (2)	−0.004 (2)	−0.0319 (18)	−0.0003 (19)
C6	0.116 (4)	0.042 (3)	0.156 (4)	−0.002 (2)	−0.082 (3)	0.006 (3)
C7	0.134 (4)	0.079 (4)	0.135 (4)	−0.059 (3)	−0.091 (3)	0.049 (3)
C8	0.094 (3)	0.088 (3)	0.099 (3)	−0.050 (3)	−0.047 (2)	0.043 (2)
C9	0.058 (2)	0.057 (2)	0.085 (2)	−0.0246 (17)	−0.0236 (17)	0.0221 (17)
C10	0.071 (2)	0.058 (2)	0.097 (3)	−0.016 (2)	−0.011 (2)	0.019 (2)
C11	0.056 (2)	0.064 (3)	0.088 (2)	−0.021 (2)	0.0027 (17)	0.0080 (19)
C12	0.059 (2)	0.058 (2)	0.0521 (17)	−0.0357 (17)	−0.0021 (14)	0.0015 (15)
C13	0.056 (2)	0.059 (2)	0.0558 (18)	−0.0344 (17)	−0.0035 (14)	−0.0080 (15)
C14	0.064 (2)	0.081 (3)	0.065 (2)	−0.035 (2)	−0.0042 (16)	−0.0037 (17)
C15	0.060 (2)	0.107 (3)	0.083 (3)	−0.034 (2)	0.0069 (19)	−0.016 (2)
C16	0.055 (2)	0.086 (3)	0.123 (3)	−0.018 (2)	−0.010 (2)	−0.016 (3)
C17	0.069 (3)	0.072 (3)	0.094 (3)	−0.025 (2)	−0.020 (2)	0.008 (2)
C18	0.068 (2)	0.064 (2)	0.064 (2)	−0.0339 (19)	−0.0061 (16)	0.0019 (17)
C19	0.066 (2)	0.055 (2)	0.101 (3)	−0.0134 (19)	−0.0087 (19)	0.0177 (19)
C20	0.050 (2)	0.062 (2)	0.083 (2)	−0.0173 (18)	0.0016 (16)	0.0118 (18)
C21	0.057 (2)	0.059 (2)	0.0456 (16)	−0.0307 (17)	−0.0069 (13)	0.0079 (14)
C22	0.0530 (19)	0.058 (2)	0.0516 (17)	−0.0299 (17)	−0.0055 (14)	−0.0022 (15)
C23	0.066 (2)	0.095 (3)	0.0609 (19)	−0.044 (2)	−0.0032 (16)	0.0005 (18)
C24	0.065 (3)	0.125 (4)	0.078 (2)	−0.046 (3)	0.0149 (19)	−0.022 (2)
C25	0.056 (2)	0.107 (4)	0.110 (3)	−0.027 (2)	0.005 (2)	−0.035 (3)
C26	0.063 (3)	0.069 (3)	0.120 (3)	−0.022 (2)	−0.010 (2)	−0.009 (2)
C27	0.056 (2)	0.063 (2)	0.081 (2)	−0.0279 (18)	−0.0015 (17)	−0.0055 (19)
C28	0.076 (3)	0.047 (2)	0.109 (3)	−0.017 (2)	−0.031 (2)	0.032 (2)
C29	0.059 (2)	0.071 (3)	0.082 (2)	−0.030 (2)	−0.0148 (17)	0.0322 (19)
C30	0.0407 (17)	0.049 (2)	0.077 (2)	−0.0118 (14)	−0.0063 (14)	0.0159 (16)
C31	0.0390 (16)	0.0395 (18)	0.0511 (15)	−0.0099 (14)	−0.0129 (12)	0.0071 (13)
C32	0.0510 (19)	0.045 (2)	0.0669 (19)	−0.0067 (16)	−0.0083 (14)	0.0102 (15)
C33	0.065 (2)	0.041 (2)	0.100 (3)	−0.0046 (17)	−0.0170 (19)	0.0077 (18)
C34	0.0305 (15)	0.0371 (18)	0.0542 (16)	−0.0063 (13)	−0.0048 (12)	0.0033 (13)
C35	0.0461 (19)	0.0358 (19)	0.103 (2)	−0.0060 (14)	−0.0025 (16)	0.0210 (17)
C36	0.0428 (18)	0.046 (2)	0.091 (2)	−0.0145 (15)	0.0043 (15)	0.0176 (17)
O1	0.0471 (13)	0.0577 (16)	0.1129 (17)	−0.0039 (12)	0.0063 (12)	0.0224 (13)
O2	0.0428 (14)	0.0719 (18)	0.153 (2)	−0.0084 (12)	0.0064 (13)	0.0407 (15)
O3	0.0750 (18)	0.0600 (18)	0.187 (3)	−0.0273 (14)	0.0146 (17)	0.0236 (17)
O4	0.0644 (16)	0.0526 (16)	0.157 (2)	−0.0223 (13)	0.0026 (15)	0.0102 (15)
O5	0.0415 (12)	0.0481 (15)	0.0978 (15)	−0.0010 (11)	0.0055 (10)	0.0192 (12)
O6	0.0352 (13)	0.0696 (17)	0.137 (2)	−0.0074 (11)	0.0056 (12)	0.0374 (14)
Ag1	0.03255 (18)	0.0589 (2)	0.0786 (2)	−0.00828 (15)	0.00271 (14)	0.00923 (17)
Ag2	0.0825 (2)	0.0775 (2)	0.1007 (2)	−0.04617 (16)	−0.00935 (15)	0.03293 (16)
Ag3	0.0386 (2)	0.0909 (3)	0.0795 (2)	−0.00848 (19)	0.00153 (16)	−0.0079 (2)
N1	0.0682 (18)	0.059 (2)	0.0698 (16)	−0.0283 (15)	−0.0077 (13)	0.0195 (14)
N2	0.0552 (16)	0.0504 (18)	0.0674 (15)	−0.0230 (14)	0.0002 (12)	0.0116 (13)
N3	0.0310 (13)	0.0396 (15)	0.0779 (16)	−0.0079 (11)	−0.0003 (11)	0.0112 (12)
N4	0.0344 (13)	0.0409 (16)	0.0744 (15)	−0.0085 (11)	0.0008 (11)	0.0086 (12)
N5	0.0680 (19)	0.0603 (19)	0.0721 (17)	−0.0312 (15)	−0.0063 (13)	0.0172 (14)
N6	0.0583 (16)	0.0553 (18)	0.0618 (15)	−0.0250 (15)	0.0004 (12)	0.0073 (12)
N7	0.0363 (13)	0.0416 (15)	0.0731 (15)	−0.0106 (12)	−0.0039 (11)	0.0087 (12)
N8	0.0415 (14)	0.0541 (18)	0.0722 (16)	−0.0119 (12)	−0.0005 (12)	−0.0007 (13)
N9	0.0483 (17)	0.060 (2)	0.0818 (18)	−0.0153 (16)	0.0076 (13)	0.0171 (15)
N10	0.0461 (17)	0.053 (2)	0.0703 (16)	−0.0096 (15)	0.0040 (12)	0.0136 (13)

### Geometric parameters (Å, °)

**Table:**

C1—C2	1.344 (4)	C22—C27	1.392 (4)
C1—N8	1.374 (4)	C22—C23	1.393 (4)
C1—H1	0.93	C23—C24	1.367 (5)
C2—N7	1.362 (3)	C23—H23	0.93
C2—H2A	0.93	C24—C25	1.367 (5)
C3—N8	1.335 (3)	C24—H24	0.93
C3—N7	1.340 (3)	C25—C26	1.368 (5)
C3—C4	1.455 (4)	C25—H25	0.93
C4—C5	1.386 (4)	C26—C27	1.377 (4)
C4—C9	1.387 (4)	C26—H26	0.93
C5—C6	1.398 (5)	C27—H27	0.93
C5—H5	0.93	C28—C33	1.363 (4)
C6—C7	1.355 (6)	C28—C29	1.368 (4)
C6—H6A	0.93	C28—H28	0.93
C7—C8	1.353 (6)	C29—C30	1.393 (4)
C7—H7A	0.93	C29—H29	0.93
C8—C9	1.384 (4)	C30—C31	1.392 (3)
C8—H8	0.93	C30—H30	0.93
C9—H9	0.93	C31—C32	1.382 (4)
C10—C11	1.349 (4)	C31—C34	1.462 (4)
C10—N5	1.363 (4)	C32—C33	1.375 (4)
C10—H10	0.93	C32—H32	0.93
C11—N6	1.346 (4)	C33—H33	0.93
C11—H11	0.93	C34—N4	1.337 (3)
C12—N5	1.326 (4)	C34—N3	1.339 (3)
C12—N6	1.359 (3)	C35—C36	1.347 (4)
C12—C13	1.451 (4)	C35—N3	1.355 (3)
C13—C14	1.386 (4)	C35—H35	0.93
C13—C18	1.395 (4)	C36—N4	1.369 (4)
C14—C15	1.368 (5)	C36—H36	0.93
C14—H14	0.93	O1—N9	1.238 (3)
C15—C16	1.365 (5)	O2—N9	1.241 (3)
C15—H15	0.93	O3—N9	1.214 (3)
C16—C17	1.374 (5)	O4—N10	1.220 (3)
C16—H16	0.93	O5—N10	1.254 (3)
C17—C18	1.376 (4)	O6—N10	1.241 (3)
C17—H17	0.93	Ag1—N4^i^	2.095 (2)
C18—H18	0.93	Ag1—N4	2.095 (2)
C19—C20	1.359 (4)	Ag2—N1	2.104 (2)
C19—N1	1.362 (4)	Ag2—N5	2.106 (2)
C19—H19	0.93	Ag3—N8^ii^	2.090 (2)
C20—N2	1.346 (4)	Ag3—N8	2.090 (2)
C20—H20	0.93	N2—H2	0.86
C21—N1	1.328 (4)	N3—H3	0.86
C21—N2	1.356 (3)	N6—H6	0.86
C21—C22	1.455 (4)	N7—H7	0.86
			
C2—C1—N8	109.9 (3)	C23—C24—H24	120.0
C2—C1—H1	125.1	C24—C25—C26	120.0 (4)
N8—C1—H1	125.1	C24—C25—H25	120.0
C1—C2—N7	105.6 (3)	C26—C25—H25	120.0
C1—C2—H2A	127.2	C25—C26—C27	120.5 (4)
N7—C2—H2A	127.2	C25—C26—H26	119.8
N8—C3—N7	109.0 (2)	C27—C26—H26	119.8
N8—C3—C4	127.6 (3)	C26—C27—C22	120.6 (3)
N7—C3—C4	123.4 (2)	C26—C27—H27	119.7
C5—C4—C9	118.6 (3)	C22—C27—H27	119.7
C5—C4—C3	121.5 (3)	C33—C28—C29	120.7 (3)
C9—C4—C3	119.9 (3)	C33—C28—H28	119.6
C4—C5—C6	119.4 (4)	C29—C28—H28	119.6
C4—C5—H5	120.3	C28—C29—C30	120.0 (3)
C6—C5—H5	120.3	C28—C29—H29	120.0
C7—C6—C5	120.7 (4)	C30—C29—H29	120.0
C7—C6—H6A	119.7	C31—C30—C29	119.8 (3)
C5—C6—H6A	119.7	C31—C30—H30	120.1
C8—C7—C6	120.6 (4)	C29—C30—H30	120.1
C8—C7—H7A	119.7	C32—C31—C30	118.3 (3)
C6—C7—H7A	119.7	C32—C31—C34	121.7 (2)
C7—C8—C9	120.1 (4)	C30—C31—C34	120.0 (2)
C7—C8—H8	120.0	C33—C32—C31	121.6 (3)
C9—C8—H8	120.0	C33—C32—H32	119.2
C8—C9—C4	120.7 (3)	C31—C32—H32	119.2
C8—C9—H9	119.7	C28—C33—C32	119.5 (3)
C4—C9—H9	119.7	C28—C33—H33	120.2
C11—C10—N5	109.6 (3)	C32—C33—H33	120.2
C11—C10—H10	125.2	N4—C34—N3	109.0 (2)
N5—C10—H10	125.2	N4—C34—C31	127.2 (2)
N6—C11—C10	106.1 (3)	N3—C34—C31	123.8 (2)
N6—C11—H11	127.0	C36—C35—N3	106.1 (2)
C10—C11—H11	127.0	C36—C35—H35	126.9
N5—C12—N6	108.5 (3)	N3—C35—H35	126.9
N5—C12—C13	127.8 (3)	C35—C36—N4	109.4 (2)
N6—C12—C13	123.7 (3)	C35—C36—H36	125.3
C14—C13—C18	118.3 (3)	N4—C36—H36	125.3
C14—C13—C12	121.2 (3)	N4^i^—Ag1—N4	180
C18—C13—C12	120.5 (3)	N1—Ag2—N5	175.94 (11)
C15—C14—C13	120.5 (3)	N8^ii^—Ag3—N8	180
C15—C14—H14	119.8	C21—N1—C19	106.7 (2)
C13—C14—H14	119.8	C21—N1—Ag2	128.9 (2)
C16—C15—C14	121.0 (3)	C19—N1—Ag2	124.3 (2)
C16—C15—H15	119.5	C20—N2—C21	108.8 (3)
C14—C15—H15	119.5	C20—N2—H2	125.6
C15—C16—C17	119.6 (4)	C21—N2—H2	125.6
C15—C16—H16	120.2	C34—N3—C35	109.1 (2)
C17—C16—H16	120.2	C34—N3—H3	125.4
C16—C17—C18	120.2 (3)	C35—N3—H3	125.4
C16—C17—H17	119.9	C34—N4—C36	106.4 (2)
C18—C17—H17	119.9	C34—N4—Ag1	129.74 (18)
C17—C18—C13	120.4 (3)	C36—N4—Ag1	123.83 (18)
C17—C18—H18	119.8	C12—N5—C10	106.9 (2)
C13—C18—H18	119.8	C12—N5—Ag2	130.1 (2)
C20—C19—N1	109.5 (3)	C10—N5—Ag2	122.3 (2)
C20—C19—H19	125.3	C11—N6—C12	108.9 (3)
N1—C19—H19	125.3	C11—N6—H6	125.5
N2—C20—C19	106.0 (3)	C12—N6—H6	125.5
N2—C20—H20	127.0	C3—N7—C2	109.3 (2)
C19—C20—H20	127.0	C3—N7—H7	125.4
N1—C21—N2	109.0 (3)	C2—N7—H7	125.4
N1—C21—C22	127.8 (3)	C3—N8—C1	106.2 (2)
N2—C21—C22	123.2 (3)	C3—N8—Ag3	129.2 (2)
C27—C22—C23	117.4 (3)	C1—N8—Ag3	124.61 (19)
C27—C22—C21	120.9 (3)	O3—N9—O1	121.0 (3)
C23—C22—C21	121.6 (3)	O3—N9—O2	121.6 (3)
C24—C23—C22	121.5 (3)	O1—N9—O2	117.4 (3)
C24—C23—H23	119.2	O4—N10—O6	122.3 (3)
C22—C23—H23	119.2	O4—N10—O5	120.1 (3)
C25—C24—C23	120.0 (3)	O6—N10—O5	117.6 (3)
C25—C24—H24	120.0		

Symmetry codes: (i) −*x*, −*y*, −*z*+2; (ii) −*x*+1, −*y*+1, −*z*+1.

### Hydrogen-bond geometry (Å, °)

**Table:**

*D*—H···*A*	*D*—H	H···*A*	*D*···*A*	*D*—H···*A*
N2—H2···O6^i^	0.86	2.03	2.893 (3)	178
N3—H3···O5^iii^	0.86	1.95	2.812 (3)	178
N6—H6···O2^ii^	0.86	1.99	2.846 (3)	173
N7—H7···O1^iv^	0.86	1.95	2.812 (3)	177

Symmetry codes: (i) −*x*, −*y*, −*z*+2; (iii) −*x*+1, −*y*, −*z*+2; (ii) −*x*+1, −*y*+1, −*z*+1; (iv) −*x*, −*y*+1, −*z*+1.
